# The Contribution of Efflux Pumps in *Mycobacterium abscessus* Complex Resistance to Clarithromycin

**DOI:** 10.3390/antibiotics8030153

**Published:** 2019-09-18

**Authors:** Júlia S. Vianna, Diana Machado, Ivy B. Ramis, Fábia P. Silva, Dienefer V. Bierhals, Michael Andrés Abril, Andrea von Groll, Daniela F. Ramos, Maria Cristina S. Lourenço, Miguel Viveiros, Pedro E. Almeida da Silva

**Affiliations:** 1 Núcleo de Pesquisas em Microbiologia Médica, Universidade Federal de Rio Grande, Rua Visconde de Paranaguá, 102, Rio Grande 96200-190, RS, Brazil; jusvianna@hotmaiL.com (J.S.V.); ivybramis@gmail.com (I.B.R.); fabiapeixoto@gmail.com (F.P.S.); dienefer_bierhals@hotmail.com (D.V.B.); andresabrilg94@gmail.com (M.A.A.); avongrol@hotmail.com (A.v.G.); daniferamos@gmail.com (D.F.R.); pedrefurg@gmail.com (P.E.A.d.S.); 2 Unidade de Microbiologia Médica, Global Health and Tropical Medicine, Instituto de Higiene e Medicina Tropical, Universidade NOVA de Lisboa, 1349-008 Lisboa, Portugal; diana@ihmt.unl.pt; 3 Instituto Nacional de Infectologia Evandro Chagas, Fundação Oswaldo Cruz, Campus Manguinhos, Rio de Janeiro 21040-360, Brazil; cristina.lourenco@ini.fiocruz.br

**Keywords:** efflux inhibitors, efflux pumps, *erm*(41), mutations, mycobacteria, verapamil

## Abstract

The basis of drug resistance in *Mycobacterium abscessus* is still poorly understood. Nevertheless, as seen in other microorganisms, the efflux of antimicrobials may also play a role in *M. abscessus* drug resistance. Here, we investigated the role of efflux pumps in clarithromycin resistance using nine clinical isolates of *M. abscessus* complex belonging to the T28 *erm*(41) sequevar responsible for the inducible resistance to clarithromycin. The strains were characterized by drug susceptibility testing in the presence/absence of the efflux inhibitor verapamil and by genetic analysis of drug-resistance-associated genes. Efflux activity was quantified by real-time fluorometry. Efflux pump gene expression was studied by RT-qPCR upon exposure to clarithromycin. Verapamil increased the susceptibility to clarithromycin from 4- to ≥64-fold. The efflux pump genes *MAB_3142* and *MAB_1409* were found consistently overexpressed. The results obtained demonstrate that the T28 *erm*(41) polymorphism is not the sole cause of the inducible clarithromycin resistance in *M.* *abscessus* subsp. *abscessus* or *bolletii* with efflux activity providing a strong contribution to clarithromycin resistance. These data highlight the need for further studies on *M. abscessus* efflux response to antimicrobial stress in order to implement more effective therapeutic regimens and guidance in the development of new drugs against these bacteria.

## 1. Introduction

Species belonging to the *Mycobacterium abscessus* complex are responsible for pulmonary and extrapulmonary infections affecting mostly the skin and soft-tissues [1,2]. These species are estimated to account for 5–20% of all nontuberculous mycobacteria (NTM) infections worldwide [3]. Nevertheless, the incidence is likely underestimated due to the absence of compulsory notification in most countries. The occurrence of outbreaks of *M. abscessus* (*sensu lato*) infections has been related to healthcare settings and occurs mainly in immunocompromised patients [3,4].

*M. abscessus* complex comprises three subspecies: *M. abscessus* subsp. *abscessus*, *M. abscessus* subsp. *massiliense* and *M. abscessus* subsp. *bolletii* [1,5]. The pattern of drug susceptibility differs according to the subspecies stressing the importance of their differentiation among the complex, as they will respond differently to the therapeutic regimen [1,6,7,8]. Therapeutic regimens are based on a macrolide, usually clarithromycin, an aminoglycoside, generally amikacin, and a β-lactam, imipenem or cefoxitin [9,10]. *M. abscessus* subspecies differ on their antimicrobial susceptibilities, particularly to macrolides, the first-line antibiotic for the treatment of these infections [10].

Macrolide resistance in *M. abscessus* can be due to mutations in the gene *erm*(41), encoding the erythromycin ribosome methyltransferase Erm(41), and to mutations in the gene *rrl*, encoding the 23S rRNA. *M. abscessus* subsp. *massiliense* has a non-functional Erm(41) [6,11,12]. Consequently, this gene is not associated with clarithromycin resistance in this subspecies. On the contrary, *M. abscessus* subsp. *abscessus* and *M. abscessus* subsp. *bolletii* have a functional Erm(41) protein. Mutations in the *erm*(41) gene are the most common mechanism of resistance to clarithromycin in these subspecies [6,12,13,14]. Resistance to aminoglycosides is mainly associated with mutations in the *rrs* gene, encoding the 16S rRNA or aminoglycoside-modifying enzymes [15] and resistance to β-lactams occurs due to the production of Bla_Mab_, a chromosome-encoded Ambler class A β-lactamase [16].

Poor treatment outcomes of *M. abscessus* infections have been attributed to innate and acquired drug resistance. Current treatment options are suboptimal and therapeutic options are limited [17,18]. *M. abscessus* is intrinsically resistant to several classes of antibiotics mainly due to the impermeability of its cell membrane. Efflux activity has been proposed as a drug resistance mechanism in mycobacteria where the induction of efflux pumps due to the exposure to non-inhibitory concentrations of antibiotics is proposed as the first step of acquired drug resistance that leads to high-level chromosomal-mutation-related resistance that may hamper the activity of several antibiotics [19,20]. Recently, efflux activity has been correlated with clinical resistance to macrolides, aminoglycosides, bedaquiline, and clofazimine also in *M. abscessus* [21,22,23]. Therefore, an alternative approach to overcome *M. abscessus* drug resistance driven by efflux activity is the inhibition of efflux of antibiotics with the aid of efflux inhibitors, compounds that are able to reduce the intrinsic resistance of the bacteria by increasing the intracellular concentration of antimicrobial compounds [23]. Real-time fluorometric assays have been used to detect and quantify increased efflux activity using fluorescent substrates, such as ethidium bromide, which is capable of accumulating inside the bacteria in the presence of an efflux inhibitor and this increased efflux activity has been correlated with clinically relevant antibiotic resistance [20,24,25,26,27].

The increasing number of infections caused by *M. abscessus*, together with the reduced therapeutic options available, highlights the need to increase our knowledge on the mechanisms of drug resistance in *M. abscessus*. Previous studies have shown that the inhibition of efflux activity by verapamil in *M. tuberculosis* and *M. avium* complex can improve the activity of antimicrobial compounds [20,28,29,30]. In this study, we have evaluated the contribution of efflux to *M. abscessus* clarithromycin resistance correlating the levels of resistance, in the presence and absence of verapamil, with the presence of mutations in the clarithromycin resistance-associated genes, *erm*(41) and *rrl*. Furthermore, we have investigated the role of increased efflux pump gene mRNA levels and the consequently increased efflux activity in six *M. abscessus* strains when exposed to sub-inhibitory concentrations of clarithromycin as one of the mechanisms contributing to inducible macrolide resistance. 

## 2. Results

### 2.1. Phenotypic and Genotypic Characterization of the M. abscessus Strains

In this work, we studied four *M. abscessus* subsp. *abscessus* strains (three clinical isolates and the ATCC19977^T^ reference strain) plus three *M. abscessus* subsp. *bolletii* and three *M. abscessus* subsp. *massiliense* clinical isolates from two geographically distinct origins, Brazil and Portugal. We used the GenoType NTM-DR assay to determine the *erm*(41) sequevars and search for mutations in the *rrl* and *rrs* genes (Table 1). We found that all the strains belong to the *erm*(41) T28 sequevar.

Clarithromycin and amikacin MICs are presented in Table 1. None of the strains presented a multidrug-resistant phenotype, i.e., simultaneous resistance to clarithromycin and amikacin [14]. With regard to amikacin, all strains were susceptible to this antibiotic (breakpoint for resistance: MIC ≥ 64 μg/mL) [10]. The susceptibility results were 100% concordant with those obtained with the GenoType NTM-DR, i.e., no mutation was detected in the *rrs* gene associated with amikacin resistance. Concerning clarithromycin, the results matched 100% with the results obtained with the GenoType NTM-DR. Nine strains were susceptible to clarithromycin and one was resistant. The latter presented an MIC of 256 μg/mL consistent with the presence of a mutation in the *rrl* gene (MIC ≥ 256 μg/mL) [10].

Next, we evaluated the presence of inducible clarithromycin resistance since all strains belong to the *erm*(41) T28 sequevar (Table 2). According to the CLSI guidelines, macrolide inducible resistance is defined as an increase in the MIC of clarithromycin from ≤2 μg/mL at day 5 to ≥8 μg/mL at day 14 of incubation [10]. Of the 10 strains tested, only three presented an inducible resistance phenotype to clarithromycin, namely two *M. abscessus* subsp. *abscessus* strains (ATCC19977^T^ and MabPT6) and one *M. abscessus* subsp. *bolletii* (MabPT2). No inducible resistance was found in the *M. abscessus* subsp. *massiliense* isolates, as expected, since this subspecies harbors a non-functional Erm(41) protein.

Of the 10 strains studied, two *M. abscessus* subsp. *bolletii* strains (MabBR2 and MabPT4) presented increased resistance to clarithromycin after five days of incubation (Table 2) that, according to the CLSI guidelines, could not be attributed to the presence of T28 nucleotide change, a finding indicating the existence of another mechanism of resistance to clarithromycin. Since no mutations were found in the *rrl* gene and active efflux has been associated with clarithromycin resistance in several microorganisms including mycobacteria and *M. abscessus* [19,20,21,22,23,30,31], we continued the work with the evaluation of the presence of increased active efflux systems despite the presence of the T28 polymorphism. For this purpose, we studied all strains to assess the contribution of active efflux to their macrolide susceptibility phenotype.

### 2.2. Evaluation of the Synergistic Effect between Efflux Inhibitors and Clarithromycin

For these assays, we selected verapamil as reference efflux inhibitor since several works have already shown that this is the most efficient efflux inhibitor for several mycobacterial species [20,23,28,29]. The MICs of verapamil are given in Table 1. The synergistic effect between verapamil and clarithromycin and the modulation factor (MF) obtained for each combination are listed in Table 3.

As can be observed, the MIC of clarithromycin at day 3 and day 5 was significantly reduced in seven of the 10 strains studied. Among the three remaining strains, MabBR1 and MabPT5 were susceptible to clarithromycin and they do not present inducible clarithromycin resistance. Their MICs decreased only two-fold in the presence of verapamil, which was not considered significant, and this phenotype was maintained until day 14 of incubation. The third strain, MabBR3, harbors a mutation in the *rrl* gene, which confers high-level resistance to clarithromycin and as expected no effect was observed on the MIC of clarithromycin in the presence of verapamil. For the remaining seven strains, the MICs of clarithromycin decreased in the presence of ½ the MIC of verapamil (determined for each strain and presented in Table 1) by four-fold to ≥32-fold at day 3 and by four-fold to ≥64-fold at day 5 (Table 3). At day 14, MabPT1 was the only strain for which the MIC of clarithromycin was reduced by four-fold in the presence of verapamil. Moreover, the lack of effect of the efflux inhibitor verapamil at day 14 is interpreted as the result of the emergence of other resistance mechanisms during clarithromycin exposure, e.g., the development of mutations in target genes associated with clarithromycin resistance such as the *rrl*, *rplV* or *rplD,* which will be analyzed in further studies. These results showed that the synergistic effect of verapamil does not depend on the *M. abscessus* subspecies studied nor by the presence of the T28 polymorphism in *erm*(41). This data demonstrates the existence of efflux activity in these strains that can be reduced by verapamil. Altogether, these results reveal, for the first time, that the inducible clarithromycin resistance in the strains assayed is also due to increased efflux activity in spite of the inducible resistance via *erm*(41).

### 2.3. Assessment of Efflux Activity by Real-Time Fluorometry

**Figure 1:**
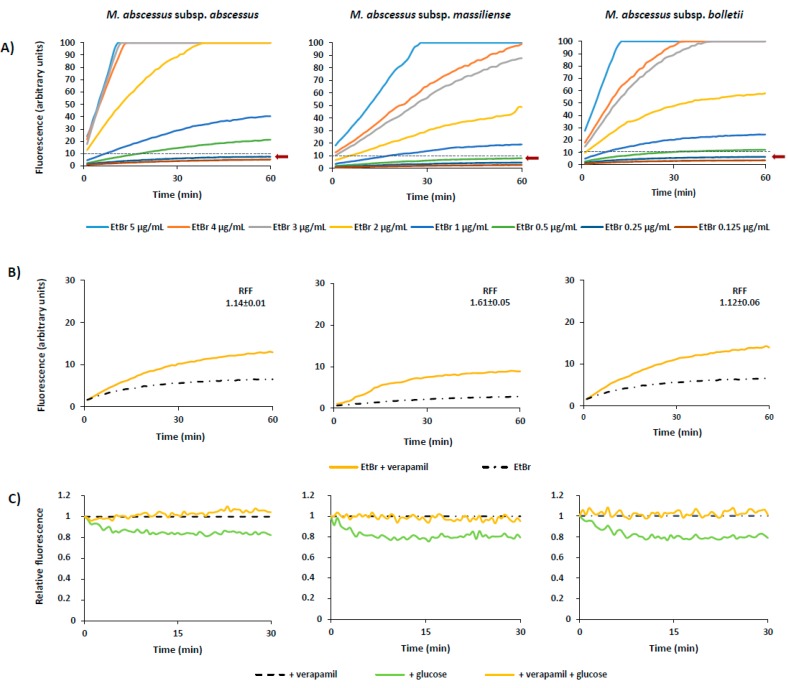
Accumulation and efflux of *M. abscessus*. The figure shows one representative assay for each subspecies, as follows: *M*. *abscessus* subsp. *abscessus*—MabPT1, *M. abscessus* subsp. *massiliense*—MabPT3, and *M. abscessus* subsp. *bolletii*—MabBR2. (**A**) Accumulation of increasing concentrations of ethidium bromide. The equilibrium concentration was determined for each strain as the concentration that promoted a plateau of no more than 10% of relative fluorescence units during the 60 min of the assay (black broken line) and is indicated in each graph by an arrow. The assays were performed in the presence of 0.4% of glucose. (**B**) Accumulation of ethidium bromide in the presence of verapamil. Each strain was tested at its equilibrium concentration (Table 4) in the presence and absence of ½ MIC of verapamil (see Table 1 for MICs). The assays were performed in the presence of glucose. RFF, relative final fluorescence. (**C**) Efflux of ethidium bromide. The strains were loaded with ethidium bromide at the equilibrium concentration and efflux took place in the presence of glucose, which was inhibited by verapamil at ½ MIC.

After the demonstration of the synergistic effect between the efflux inhibitor verapamil and clarithromycin, indirectly correlating efflux with resistance via the inhibitory effect of verapamil, we studied the presence of active efflux activity in these strains by real-time fluorometry using the broad efflux substrate ethidium bromide in the presence of glucose as a source of metabolic energy and in the presence and absence of verapamil. First, was determined the lowest concentration that causes minimal accumulation of ethidium bromide, i.e., the concentration for which there is an equilibrium between influx and efflux of the substrate (Figure 1A, Table 4).

The lowest concentration that resulted in an equilibrium between the influx and efflux of ethidium bromide (Ceq) for all the strains varied between 0.125 μg/mL to 0.5 μg/mL (Table 4).

Next, we performed ethidium bromide accumulation assays in the presence of verapamil as a proof of concept (Figure 1B) and determined the RFF indexes (Table 4). The RFF index reflects how efficient verapamil is in promoting intracellular accumulation of ethidium bromide, in this case, in *M. abscessus*. The results showed that verapamil is able to increase the ethidium bromide accumulation in all the tested strains (Table 4) and at highly significant levels in six of the 10 strains studied. The RFF values of these strains are in the same range varying between 1.12 to 1.64. Comparing the different subspecies, no relevant differences were observed on the levels of ethidium bromide accumulation. These results demonstrate that verapamil is able to promote intracellular accumulation of ethidium bromide on *M. abscessus* evidencing that real-time active efflux is present in this species and can be inhibited by verapamil.

To confirm that the efflux activity of these strains could be inhibited in the presence of verapamil we performed efflux assays (Figure 1C). Analyzing the results of each strain and comparing the curves corresponding to the strain in the presence of verapamil and glucose (orange) with the curve of the strain in the presence of glucose but without verapamil (green), we conclude that ethidium bromide efflux is inhibited by verapamil in the *M. abscessus* strains studied, strengthening the correlation between active efflux and drug resistance in these strains.

### 2.4. Quantification of Efflux Pump mRNA Levels by RT-qPCR

**Figure 2:**
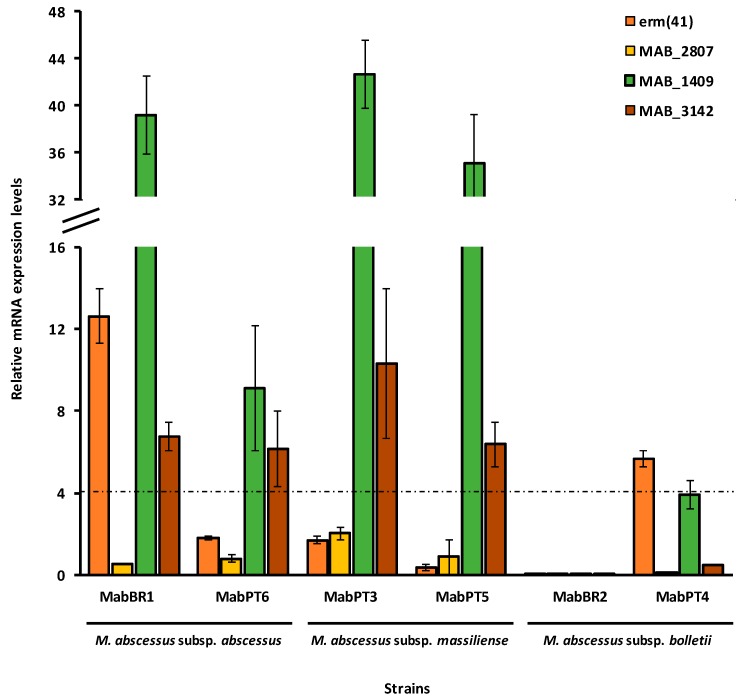
Relative quantification of efflux pump gene mRNA levels in the *M. abscessus* strains exposed to clarithromycin. Strains were grown in MGIT medium for the MGIT 960 system in the presence of half MIC of clarithromycin (see Table 1 for MICs). The relative expression of the efflux pump genes was evaluated comparing the relative quantity of the respective mRNA in the presence of clarithromycin to the respective unexposed strain. A level of relative expression equal to 1 indicates that the expression level is identical to the non-exposed parental strain. Genes showing expression levels above four were considered overexpressed (black dashed line in the graph).

To analyze the contribution of the overexpression of efflux pumps to clarithromycin resistance, we selected three main efflux pump genes correlated with antibiotic-resistant phenotypes, including macrolides, in mycobacteria and in particular in *M. tuberculosis*, as proof of concept [20,23,32], namely *MAB_3142* (homologue of *M. tuberculosis p55*), *MAB_1409* (*Rv1258c* or *tap-like*) and *MAB_2807* (*efpA*). We chose two strains of each *M. abscessus* subspecies to analyze the changes in the mRNA transcriptional levels after exposure to ½ of the respective MIC of clarithromycin. The relative mRNA transcription levels were determined against the respective non-exposed strain (Figure 2).

Increased mRNA levels were detected in all strains with the sole exception of MabBR2. This strain does not present overexpression of any of the three efflux pump genes studied nor *erm*(41). Comparing these results with those obtained in the MICs assays, we noticed that MabBR2 was susceptible to clarithromycin at day 3, with an MIC of 2 μg/mL. The concentration used for the analysis of efflux pump mRNA levels was 1 μg/mL. Although there was a four-fold decrease in the MIC of clarithromycin in the presence of verapamil at day 3, this effect may be attributed to a combined effect between the two compounds and not exclusively to efflux pump overexpression at this time point. Moreover, MabBR2 does not have inducible clarithromycin resistance which is consistent with a non-expressed *erm*(41) gene. Notably, at day 5 we noted a reversion of the phenotype from resistant to susceptible in the presence of verapamil that may be attributed to the efflux activity due to the prolonged exposure to clarithromycin. At day 14, no effect was seen in the presence of the efflux inhibitor, indicating that the *M. abscessus* subsp. *bolletii* BR2 may have acquired other resistance mechanisms as a mutation in the *rrl* gene that is associated with high-level clarithromycin resistance (MIC ≥ 256 μg/mL) [10]. Therefore, we studied another *M. abscessus* subsp. *bolletii* strain (MabPT4) to assess the contribution of the subspecies to the response to clarithromycin exposure. In terms of antibiotic susceptibility in the presence and absence of verapamil, the results are entirely equivalent between both strains (Table 3), however, MabPT4 has the *erm*(41) gene overexpressed despite having a non-inducible phenotype and shows borderline overexpression of the *MAB_1409* (*tap-like*) gene (Figure 2). This result shows that *erm*(41) gene expression is not the sole cause of clarithromycin resistance in this strain. Efflux activity is present in this strain (Table 4) representing a late stress response to clarithromycin exposure. Concerning *M. abscessus* subsp. *massiliense*, we analyzed MabPT3 and MabPT5, both susceptible to clarithromycin without inducible resistance consistent with the lack of expression of *erm*(41) encoding a non-functional Erm(41) protein. Although susceptible, the MICs of clarithromycin decreased in the presence of verapamil by four-fold in MabPT3 and two-fold in MabPT5 (Table 3). Both strains showed overexpression of *MAB_3142* and *MAB_1409* (Figure 2). Comparing to the real-time efflux assays, MabPT3 has increased efflux activity although MabPT5 showed lower efflux activity than MabPT3. This result shows that efflux activity acts also as a first-line response in these *M. abscessus* subsp. *massiliense* strains. Finally, we studied two strains of *M. abscessus* subsp. *abscessus*, namely MabBR1 and MabPT6. MabBR1 showed intermediate resistance at day 14, and consequently, non-inducible resistance to clarithromycin, whereas MabPT6 was resistant at day 14 through an inducible resistant phenotype to this antibiotic. Comparing this data with the RT-qPCR assays, MabBR1 exhibited overexpression of *MAB_3142* and *MAB_1409* efflux pump genes and, somehow unexpected, also *erm*(41). On the contrary, MabPT6 that presents inducible resistance to clarithromycin, although at low levels (16 µg/mL), showed no expression of *erm*(41). However, this strain shows overexpression of *MAB_3142* and *MAB_1409* efflux pump genes. Comparing with the real-time efflux assays, MabBR1 showed lower levels of efflux than MabPT6, probably due to the presence of an overexpressed *erm*(41). No overexpression was observed concerning the gene *MAB_2807* for any of the six strains analyzed.

## 3. Discussion

Members of the *M. abscessus* complex are clinically important pathogens. At present, only few antibiotics showed antimicrobial activity with clinical significance against this species, namely, clarithromycin and amikacin, and either cefoxitin or imipenem. The main reasons commonly accepted for this scarce therapeutic panel is the low permeability of *M. abscessus* complex cell wall, the presence of mutations in the drug target genes and, in the case of clarithromycin, the presence of an inducible *erm*(41) gene [14]. Recently, the hypothesis of efflux-mediated drug resistance has been appointed as a putative contributor to drug resistance in *M. abscessus* [21,22].

All bacteria express efflux pumps. Efflux transporters are transmembrane proteins involved in the extrusion of harmful compounds and cellular metabolites from the cells into the external environment [33]. Indirectly, they are also involved in drug resistance by expelling antimicrobials, reducing their intracellular concentration. Treatment failure has been mainly attributed to *M. abscessus* belonging to the T28 sequevar [12,34,35]. Similar to what is observed in *M. avium* complex infections, acquired resistance to clarithromycin also evolves rapidly during therapy in *M. abscessus* [36]. Therefore, it is legitimate to raise the hypothesis of whether the overexpression of efflux pumps is also involved in the resistance to clarithromycin even in the *M. abscessus* sequevar T28? Ongoing work showed that resistance towards clarithromycin in *M. avium* results from the balance between the presence of drug resistance-associated mutations and increased efflux activity. We also noticed that this increased efflux activity was accompanied by the overexpression of several efflux pump genes upon exposure to clarithromycin. Therefore, to answer this question, we investigated the contribution of efflux transporters to the induced clarithromycin resistance in *M. abscessus* complex belonging to the *erm*(41) T28 sequevar.

The strains were blindly selected in relation to their clarithromycin susceptibility status and included a panel of nine clinical strains, comprising three isolates of each *M. abscessus* subspecies, namely, subsp. *abscessus*, *bolletii* and *massiliense*. The *M. abscessus* subsp. *abscessus* ATCC19977^T^ reference strain was included as control. Firstly, we searched for mutations associated with clarithromycin resistance the *M. abscessus* strains. We found that all belong to the *erm*(41) T28 sequevar which, at least theoretically, is responsible for the inducible resistance to clarithromycin in *M. abscessus* [12,14]. One of the strains harbors a mutation in *rrl*, and consequently, displays high-level resistance to clarithromycin, and was used as mutation-driven resistance control. These initial results are in accordance with those described in the literature that states that the majority of the *M. abscessus* strains are susceptible to clarithromycin and resistance is acquired during treatment [1,14]. To test the role of the *erm*(41) T28 polymorphism in the induced resistance of the strains analyzed, we read the MICs at day 5 of incubation and prolonged the incubation until day 14. Surprisingly, of the eight strains, only two clinical strains and the reference strain showed induced resistance to clarithromycin, one *M. abscessus* subsp. *abscessus* and the other *M. abscessus* subsp. *bolletii*. Interestingly, two *M. abscessus* subsp. *bolletii* strains were shown to be already resistant to clarithromycin at day 5 and according to the CLSI guidelines, they do not fit in the category of clarithromycin-inducible resistance. These results demonstrate that the T28 polymorphism in *erm*(41) is not the sole cause of clarithromycin-induced resistance in these *M. abscessus* strains indicating the existence of another mechanism of resistance in this collection of strains.

We then studied the presence of active efflux activity in these strains through the determination of the MICs of clarithromycin in the presence of the efflux inhibitor verapamil. The results obtained supported our hypothesis that efflux is also involved in *M. abscessus* induced resistance to clarithromycin. At day 3 and day 5, all the MICs of clarithromycin for all strains, with the sole exception of the strain with the *rrl* mutation, could be reduced in the presence of the efflux inhibitor verapamil. In particular, it was possible to reverse to clinically significant levels the resistance to clarithromycin at day 5 for four strains, one from resistant to susceptible, one from resistant to intermediate and two from intermediate to susceptible. After 14 days of exposure, no inhibitory effect was noted for these, meaning that these strains may have acquired other mutational mechanisms of resistance, a subject that will be addressed in subsequent studies. Concurrently with real-time fluorometric assays, using verapamil as reference efflux inhibitor and ethidium bromide as substrate, we detected significant efflux activity in the 10 strains, which could be inhibited in the presence of verapamil. It was also demonstrated that this efflux activity does not depend on subspecies of the strains, as it was detected in all the three subspecies, although at different levels, nor due to their phenotype since it was detected in inducible and non-inducible clarithromycin resistance strains. These data demonstrated the presence and importance of active efflux in *M. abscessus* strains resistant to clarithromycin, which could be inhibited by verapamil.

Finally, to further analyze the contribution of the expression of efflux pumps to clarithromycin resistance in these strains, three efflux pump genes, *MAB_3142*, *MAB_1409* and *MAB_2807*, and the *erm*(41) gene, were selected to examine changes in mRNA transcriptional levels after exposure to ½ MIC of clarithromycin determined for each of the six strains selected. We noted increased mRNA expression levels of the efflux pumps *MAB_3142* and *MAB_1409* in four out of six of the strains and found also *erm*(41) overexpression in two strains, one presenting inducible resistance while the other does not have induced resistance to clarithromycin.

This exploratory study consolidates and strengthens the hypothesis that efflux activity plays a role in *M. abscessus* intrinsic resistance to clarithromycin in strains belonging to the T28 sequevar and that the inhibition of this efflux activity by compounds such as verapamil could potentiate the clinical effect of clarithromycin, the most efficient antibiotic to treat *M. abscessus* infections, certainly reducing or preventing the emergence of the, so far almost inexorable, acquired resistance to macrolides during treatment against *M. abscessus* infections.

## 4. Materials and Methods

### 4.1. M. abscessus Complex Strains

The strains selected for this study are described in Table 1 and were isolated from Brazilian (BR) and Portuguese (PT) patients. Brazilian clinical strains were granted by Fundação Oswaldo Cruz (Rio de Janeiro, Brazil). The Portuguese clinical strains are part of the strain collection of the Mycobacteriology Laboratory of the Instituto de Higiene e Medicina Tropical, Universidade NOVA de Lisboa (Lisboa, Portugal). The strains were identified using the GenoType NTM-DR assay (Hain Lifescience, GmbH, Nehren, Germany) according to the manufacturer’s instructions. Informed consent was not required for the present study since it corresponds to a retrospective study from which all patient identification was unlinked from the results, and no patient information was collected. *M. abscessus* subsp. *abscessus* ATCC19977^T^ reference strain was obtained from the American Type Culture Collection (Virginia, USA) and was included as a control strain.

### 4.2. Compounds and Reagents

Amikacin, clarithromycin, verapamil, ethidium bromide, dimethyl sulfoxide (DMSO), and glucose were purchased from Sigma-Aldrich (St. Louis, MO, USA). Clarithromycin was prepared in DMSO, while the remaining drugs were prepared in sterile deionized water. Stock solutions were stored at −20 °C. Working solutions of both antibiotics and ethidium bromide were prepared in Mueller–Hinton broth (MHB; Oxoid, Hampshire, UK) on the day of the experiments. Löwenstein–Jensen medium, Middlebrook 7H9, BBL OADC (oleic acid/albumin/dextrose/catalase) supplement and the mycobacteria growth indicator tubes (MGITs) were purchased from Becton Dickinson (Diagnostic Systems, Sparks, MD, USA).

### 4.3. Detection of Mutations Associated with Clarithromycin and Amikacin Resistance

Total DNA was extracted from clinical strains and the reference strain using the GenoLyse kit (Hain Lifescience) according to the manufacturer’s instructions. The presence of mutations in *rrl*, *erm*(41) and *rrs* were analyzed with the Genotype NTM-DR assay.

### 4.4. Susceptibility Testing

#### 4.4.1. Growth of the Strains

All strains were maintained in MGITs tubes supplemented with 10% OADC. For minimum inhibitory concentration (MIC) determination and synergism assays, the *M. abscessus* strains were inoculated in Löwenstein–Jensen medium and incubated at 37 °C for three to five days.

#### 4.4.2. MIC Determination of Compounds

For the determination of the MICs of the antibiotics, the efflux inhibitor verapamil and the efflux substrate ethidium bromide, the inoculum of each strain was prepared by diluting the bacterial cultures in MHB to a final density of approximately 10^5^ cells/mL [37]. The MICs were determined using a tetrazolium microplate-based assay (TEMA) [38] with small modifications. Briefly, aliquots of 0.1 mL of inoculum were transferred to each well of the plate that contained 0.1 mL of each compound at concentrations prepared from two-fold serial dilutions in MHB medium. Growth controls (only inoculum without drug) and a sterility control (only medium) were included in each plate. Two hundred microliters of sterile deionized water were added to the outer-perimeter wells of the 96-well plates to reduce evaporation of the medium during the incubation. The inoculated plates were sealed in permeable plastic bags and incubated at 37 °C for three days. After this period, 10 µL of a solution of 5× MTT/10%T80 (1:1) was added to each well and the plates incubated for four hours at room temperature. The bacterial growth was registered for each well based on the MTT color change. As the intensity of the color generated is directly proportional to the number of viable cells, a precipitate of cells stained black can be observed in the bottom of the well. The MIC was defined as the lowest concentration of compound that totally inhibited bacterial growth, i.e., no color change [39]. All assays were performed in triplicate. For amikacin, the breakpoints used were: ≤16 µg/mL, susceptible; 32 µg/mL, intermediate; and ≥64 µg/mL, resistant. For clarithromycin at day 3, the breakpoints used were: ≤2 µg/mL, susceptible; 4 µg/mL, intermediate and ≥8 µg/mL, resistant [10].

To evaluate clarithromycin induced resistance, the MIC determination of clarithromycin was extended for 14 days. To do this, plates containing clarithromycin were prepared in quadruplicate to be read at the following time points: 3, 5 and 14 days [14]. After each period of incubation, MTT was added and the results read and interpreted as described above. Susceptible isolates at day 3 (MIC ≤ 2 µg/mL) and resistant at day 5 (MIC ≥ 8 µg/mL), were considered to have an inducible *erm*(41) gene [10]. All assays were carried out in triplicate.

#### 4.4.3. MIC Determination of Antibiotics and Ethidium Bromide in the Presence of Verapamil

The MICs of amikacin, clarithromycin, and ethidium bromide were determined in the presence of verapamil. The assays were performed as described above with the exception that verapamil was added to each well to a final concentration of ½ of the MIC. The results were read and interpreted as described above. The modulation factor (MF) was calculated and used to quantify the inhibitory effect of verapamil on the MICs of clarithromycin through the following formula: MF = MIC antimicrobial / MIC antimicrobial + efflux inhibitor [40]. The MF reflects the reduction of the MIC values of a given antimicrobial in the presence of an efflux inhibitor, being considered significant when MF ≥ 4 (four-fold reduction) [41]. Each assay was carried out in triplicate.

#### 4.4.4. Semi-Automated Fluorometric Method

The evaluation of efflux activity on a real-time basis was performed using a semi-automated fluorometric method [26] with modifications [Machado et al., unpublished]. The *M. abscessus* strains were grown in 10 ml of 7H9/10%OADC with 0.05% tyloxapol at 37 °C until they reached an OD_600_ of 0.8. For the accumulation assays, the cultures were centrifuged at 3500 rpm for three minutes, the supernatant discarded and the pellet washed in 7H9. The OD_600_ was adjusted to 0.8 with 7H9. In order to determine the concentration of ethidium bromide that establishes the equilibrium between efflux and influx, aliquots of 0.05 mL of the bacterial suspension were added to 0.2 mL microtubes containing 0.05 mL different concentrations of ethidium bromide that ranged from 0.625 μg/mL to 5 μg/mL with and without 0.4% glucose. The assays were conducted at 37 °C in a Rotor-Gene 3000 (Corbett Research, Sydney, Australia), using the 530 nm band-pass and the 585 nm high-pass filters as the excitation and detection wavelengths, respectively. Fluorescence data were acquired every 60 seconds for 60 min. The selected concentration of ethidium bromide was further used for the evaluation of the capacity of verapamil to retain ethidium bromide inside the cells. Verapamil was tested at ½ MIC with and without 0.4% glucose and ethidium bromide at the equilibrium concentration determined for each strain and the assays performed as described above. The inhibitory activity of verapamil was determined through the determination of the relative final fluorescence (RFF) value according to the following formula [42]: RFF = (RF_treated_-RF_untreated_)/RF_untreated_. In this formula, the RF_treated_ corresponds to the relative fluorescence at the last time point of ethidium bromide accumulation curve (min 60) in the presence of verapamil, and the RF_untreated_ corresponds to the relative fluorescence at the last time point of the ethidium bromide accumulation curve of the untreated control tube. An index of activity above zero indicate that the cells accumulate more ethidium bromide under the condition used than those of the control (untreated cells). Negative RFF values indicate that the treated cells accumulated less ethidium bromide than those of the control condition [28,32].

For the efflux assays, the strains were exposed to conditions that promote the maximum accumulation of ethidium bromide, i.e., ethidium bromide equilibrium concentration for each strain, no glucose, presence of verapamil at ¼ MIC, and incubation at 20 °C for one hour [26]. Before incubation, the cultures were centrifuged at 3500 rpm for three minutes, resuspended in 7H9, centrifuged again and OD_600_ adjusted to 0.4. The suspension was incubated with ethidium bromide and verapamil under the conditions described above. Aliquots of 0.05 mL of cells were transferred to 0.2 mL microtubes containing 0.05 mL of verapamil at ½ MIC without ethidium bromide. Control tubes with only cells and cells with and without 0.4% glucose were included. Fluorescence was measured in a Rotor-Gene™ 3000 and data were acquired every 30 seconds for 30 min. Efflux activity was quantified by comparing the fluorescence data obtained under conditions that promote efflux (presence of glucose and absence of efflux inhibitor) with the data from the control in which the mycobacteria are under conditions of no efflux (presence of an inhibitor and no energy source). Thus, the relative fluorescence corresponds to the ratio of fluorescence that remains per unit of time, relatively to the ethidium bromide-loaded cells [27].

### 4.5. Efflux Pump Gene Expression

#### 4.5.1. Sample Preparation

For the analysis of efflux pump gene expression, each strain was inoculated into 7H9 containing 10% OADC plus clarithromycin at ½ the MIC (see Table 1 for MIC values). The strains were then incubated at 37 °C within the MGIT system until growth was achieved [20]. At this time point, samples of each culture were taken for RNA extraction and RT-qPCR analysis. 

#### 4.5.2. RNA Extraction

Total RNA was isolated from the cells using the RNeasy mini kit (QIAGEN, GmbH, Hilden, Germany) according to the manufacturer’s instructions with modifications [20]. Briefly, from a culture with 100–200 GU (about 10^6^–10^8^ cells/ml), 1 ml aliquot was removed and centrifuged at 13,000 rpm during 10 min. Then, 500 µL supernatant was removed and 1 mL of RNAprotect Bacteria Reagent (QIAGEN) added. An enzymatic lysis step was carried out with lysozyme at 3 mg/mL (Sigma) for 10 min, followed by lysis in an ultrasonic bath at 35 kHz (Gen-Probe, CA, USA) during 15 min. The RNA was purified using the RNeasy kit (QIAGEN) and treated with RNase-free DNase I (QIAGEN) during 2 h and 15 min by on-column digestion at room temperature to reduce the presence of contaminating DNA. All RNA samples were stored at −20 °C until required.

#### 4.5.3. RT-qPCR Assay

The relative expression level of mRNA of genes that code for membrane efflux transporters in *M. abscessus* (*MAB_2807, MAB_1409* and *MAB_3142*) and *erm*(41) was analyzed by RT-qPCR. The normalization of the data was done using the *M. abscessus* 16S rDNA. The RT-qPCR procedure was performed in a Rotor-Gene 3000 thermocycler and followed the protocol recommended for use with the One-step NZY RT-qPCR Green kit (Nzytech, Portugal). The determination of the relative mRNA expression level was performed using the comparative quantification cycle (Cq) method [43]. The relative expression of the genes analyzed was assessed by comparison of the relative quantity of the respective mRNA in the presence of clarithromycin to the non-exposed culture, following the same technical approach previously published [20]. Each culture was assayed in triplicate using total RNA obtained from three independent cultures. A level of relative expression equal to 1 indicates that the expression level was identical to the unexposed strain. Genes showing expression levels equal or above four, when compared with the unexposed strain, were considered to be overexpressed [20].

## 5. Conclusions

The results obtained in this study provide evidence that efflux activity is an intrinsic characteristic of *M. abscessus* clinical strains and is directly involved in clarithromycin resistance. Literature review indicated that *M. abscessus* subsp. *abscessus* T28 and *M. abscessus* subsp. *bolletii* T28 are intrinsically resistant to clarithromycin conferred by an altered Erm(41) methylase [1]. The change of cytosine by thymine at position T28 on Erm(41) causes resistance to clarithromycin due to prolonged exposure leading to treatment failure [12,13,14]. In our hands, the results obtained pointed out that *erm*(41) expression is not the sole cause of the inducible clarithromycin resistance in *M. abscessus* subsp. *abscessus* and *M. abscessus* subsp. *bolletii* T28 sequevars and that efflux pump overexpression has a strong contribution to clarithromycin resistance acting both as a first-line response or a late response to drug pressure. This study was designed as a proof of concept. Therefore, due to the small number of strains studied, we could not generalize the results obtained here. Future work will include a higher number of strains belonging to the three subspecies. Another limitation of this study, consequence of the random selection of the strains, is the absence of strains from the C28 sequevar, which will be also studied further. Nevertheless, our results point out efflux as an important contributor to the emergence of *M. abscessus* clarithromycin resistance and highlight the need of further studies on *M. abscessus senso lato* efflux response to stress imposed by antimicrobials in order to implement more effective therapeutic regimens and aid in the development of new drugs against these bacteria.

## Figures and Tables

**Table 1:** Phenotypic and genotypic characterization of the *M. abscessus* strains studied.

Strain	Species/Subspecies	Genetic Background			MICs (µg/mL), Day 3 *			
		CLA		AMK	Compounds			
		*erm*(41)	*rrl*	*rs*	CLA	AMK	VP	EtBr
MabATCC19977^T^	*M. abscessus* subsp. *abscessus*	T28	wt	wt	2 (S)	16 (S)	1024	128
MabBR1	*M. abscessus* subsp. *abscessus*	T28	wt	wt	2 (S)	8 (S)	4096	256
MabBR2	*M. abscessus* subsp. *bolletii*	T28	wt	wt	1 (S)	4 (S)	1024	64
MabBR3	*M. abscessus* subsp. *massiliense*	T28	A2059C	wt	256 (R)	16 (S)	1024	32
MabPT1	*M. abscessus* subsp. *abscessus*	T28	wt	wt	0.25 (S)	4 (S)	1024	64
MabPT2	*M. abscessus* subsp. *bolletii*	T28	wt	wt	2 (S)	4 (S)	2048	128
MabPT3	*M. abscessus* subsp. *massiliense*	T28	wt	wt	0.5 (S)	4 (S)	1024	256
MabPT4	*M. abscessus* subsp. *bolletii*	T28	wt	wt	2 (S)	4 (S)	1024	128
MabPT5	*M. abscessus* subsp. *massiliense*	T28	wt	wt	1 (S)	4 (S)	1024	256
MabPT6	*M. abscessus* subsp. *abscessus*	T28	wt	wt	1 (S)	4 (S)	2048	64

* Ref [10]. AMK, amikacin; CLA, clarithromycin; EtBr, ethidium bromide; MIC, minimum inhibitory concentration; R, resistant; S, susceptible; VP, verapamil; wt, wild-type sequence.

**Table 2:** Analysis of inducible vs. non-inducible clarithromycin resistance in the *M. abscessus* strains studied.

Strain	Mutations		CLA MICs (µg/mL)		CLA Susceptibility Profile at Day 14 *	
	*erm*(41)	*rrl*	Day 5	Day 14		
MabATCC19977^T^	T28	wt	4 (I)	16 (R)	Resistant	Inducible
MabBR1	T28	wt	2 (S)	4 (I)	Intermediate	Non-inducible
MabBR2	T28	wt	16 (R)	256 (R)	Resistant	Non-inducible
MabBR3	T28	A2059C	256 (R)	256 (R)	Resistant	Non-inducible
MabPT1	T28	wt	0.25 (S)	2 (S)	Susceptible	Non-inducible
MabPT2	T28	wt	4 (I)	16 (R)	Resistant	Inducible
MabPT3	T28	wt	0.5 (S)	0.5 (S)	Susceptible	Non-inducible
MabPT4	T28	wt	16 (R)	64 (R)	Resistant	Non-inducible
MabPT5	T28	wt	1 (S)	1 (S)	Susceptible	Non-inducible
MabPT6	T28	wt	2 (S)	16 (R)	Resistant	Inducible

CLA, clarithromycin; I, intermediate; MIC, minimum inhibitory concentration; R, resistant; S, susceptible; wt, wild-type sequence. * Ref. [10].

**Table 3:** Minimum inhibitory concentration and modulation factor of clarithromycin in the presence of verapamil for the *M. abscessus* strains in study.

Strain	MIC (µg/mL) (MF)					
	Day 3		Day 5		Day 14	
	CLA	CLA+VP	CLA	CLA+VP	CLA	CLA+VP
MabATCC19977^T^	2 (S)	0.5 (S) (↓4)	4 (I)	1 (S) (↓4)	16 (R)	16 (R)
MabBR1	2 (S)	1 (S) (↓2)	2 (S)	2 (S)	4 (I)	4 (I)
MabBR2	1 (S)	0.25 (S) (↓4)	16 (R)	2 (S) (↓8)	256 (R)	128 (R) (↓2)
MabBR3	256 (R)	256 (R)	256 (R)	256 (R)	256 (R)	256 (R)
MabPT1	0.25 (S)	0.0625 (S) (↓4)	0.25 (S)	0.0625 (S) (↓4)	2 (S)	0.5 (S) (↓4)
MabPT2	2 (S)	≤0.0625 (S) (≥↓32)	4 (I)	≤0.0625 (S) (≥↓64)	16 (R)	16 (R)
MabPT3	0.5 (S)	0.0625 (S) (↓4)	0.5 (S)	0.25 (S) (↓2)	0.5 (S)	0.5 (S)
MabPT4	2 (S)	0.5 (S) (↓4)	16 (R)	4 (I) (↓4)	64 (R)	32 (R) (↓2)
MabPT5	1 (S)	0.5 (S) (↓2)	1 (S)	0.5 (S) (↓2)	1 (S)	0.5 (S) (↓2)
MabPT6	1 (S)	≤0.03125 (S) (≥↓32)	2 (S)	0.125 (S) (↓16)	16 (R)	8 (R) (↓2)

CLA, clarithromycin; I, intermediate; MIC, minimum inhibitory concentration; MF, modulation factor; R, resistant; S, susceptible; VP, verapamil. In red are denoted the reductions of the MICs of clarithromycin in the presence of verapamil corresponding to a MF ≥ 4.

**Table 4:** Characterization of the *M. abscessus* strains according to their efflux capacity.

Strains	C_Eq_ (μg/mL)	RFF_VP_
MabATCC19977^T^	0.25	1.15 ± 0.03
MabBR1	0.125	0.78 ± 0.01
MabBR2	0.25	1.12 ± 0.06
MabBR3	0.125	0.19 ± 0.05
MabPT1	0.25	1.14 ± 0.01
MabPT2	0.25	0.63 ± 0.01
MabPT3	0.5	1.61 ± 0.05
MabPT4	0.125	1.61 ± 0.01
MabPT5	0.5	0.26 ± 0.00
MabPT6	0.25	1.64 ± 0.11

C_Eq_, equilibrium concentration; RFF, relative final fluorescence; VP, verapamil
